# Microbial diversity and function in bamboo ecosystems

**DOI:** 10.3389/fmicb.2025.1533061

**Published:** 2025-06-25

**Authors:** Yexuan Wang, Huimin Ren, Yue Zhong, Ruisheng Song, Siyuan Jiang, Mengjing Lai, Yuqi Shen, Shenkui Liu, Wenhui Shi, Guoning Qi

**Affiliations:** ^1^National Key Laboratory for Development and Utilization of Forest Food Resources, Zhejiang A&F University, Hangzhou, China; ^2^Provincial Key Laboratory for Non-wood Forest and Quality Control and Utilization of Its Products, Zhejiang A&F University, Hangzhou, China

**Keywords:** bamboo, endophytes, rhizosphere microorganisms, microbial composition, influence factors

## Abstract

Bamboo is widely distributed or cultivated globally, offering significant economic and ecological values. Soil microorganisms are crucial for plant environmental adaptation, playing essential roles in regulating plant growth and development, nutrient absorption, and resistance to environmental stresses. In recent years, substantial progress has been made in the study of bamboo soil microorganisms. This review highlights the scientific challenges in understanding the interactions between bamboo and soil microorganisms, summarizes the research progress, and discusses future research directions. The microbial community composition and diversity in various bamboo soils have been successfully characterized, with some bamboo-associated microorganisms identified and shown to promote plant growth, demonstrating considerable application potential. It has been established that the composition of soil microorganisms in bamboo is influenced by factors such as bamboo species, spatial and temporal distribution, tissue specificity, management practices, and symbiosis with other plants. Future research will likely focus on the functional genomics of bamboo, the screening and identification of bamboo-specific soil microbial communities, the dynamic responses of these microbes to environmental changes, and the molecular mechanisms regulating bamboo growth and environmental adaptation.

## Highlights

• Endophytes and rhizosphere microorganisms are crucial in bamboo plant life.
• Bamboo-associated microorganisms have been extensively studied through culture and high-throughput sequencing.
• Microbial composition and function related to bamboo are regulated by species and spatiotemporal factors.
• Research on bamboo-microbe interactions will advance high-quality bamboo forest cultivation.

## 1 Introduction

Plants provide a rich ecological niche for microorganisms, including bacteria, fungi, protozoa, nematodes, and viruses, which can establish complex interconnections with plants and play crucial roles in promoting plant growth, nutrient acquisition, and stress resistance (Trivedi et al., 2020). Increasing evidence suggested that plant evolution was closely linked to the microbial communities inhabiting their surroundings, with microbes acting as essential symbiotic partners that shaped plant fitness and adaptability (Vandenkoornhuyse et al., 2015). While plant-microbe interactions have been extensively studied in various model and crop plants, the specific mechanisms underlying bamboo-associated microbial communities remain relatively underexplored.

Bamboos, classified under the subfamily Bambusoideae of the Poaceae family, have become one of the most valuable plants in Poaceae family for their wide distribution, huge economic and ecological value. By 2015, they have covered over 30 million hectares worldwide (Lin, 2021). Bamboo is primarily distributed across three major global bamboo regions: the Asia-Pacific, the Americas, and the Africa, with China hosting about half of the world’s bamboo resources (Jiang, 2008). According to the 2021 China Forest and Grass Ecological Comprehensive Monitoring and Evaluation Report, bamboo forests span 7.56 million hectares across 20 provinces in China. In addition, bamboo is valued not only for its economic benefits but also for its role in carbon sequestration, soil and water conservation, and ecosystem restoration (Yang, 2004), underscoring their ecological and economic significance. However, bamboo growth is highly susceptible to environmental stressors such as extreme temperatures, drought, and soil degradation (Wang et al., 2022; Li et al., 2017), and microbes play an important role in coping with these environmental changes. By the end of 2018, 130 genera and 1,700 species of bamboo had been identified globally (Zhong et al., 2019), demonstrating its high species diversity. The microbial communities recruited by different bamboo species are different, which provides a rich research content for the study of bamboo-microbe interaction. Despite increasing recognition of plant-microbe interactions as key regulators of plant adaptation, the role of microbial communities in modulating bamboo resilience to environmental challenges remains insufficiently studied. Research on bamboo—microbe interactions have gained significant attention for its potential to enhance bamboo growth and cultivation conditions.

To address this gap, this review aims to: (1) summarize the current understanding of bamboo—associated microbial communities, including their composition, functions, and ecological roles; (2) examine how environmental and management factors influence bamboo microbiomes; and (3) identify knowledge gaps and propose future research directions in bamboo-microbe interactions. In addition, we discuss how bamboo-microbe interactions fit within the broader framework of plant-microbe symbioses, highlighting their potential contributions to sustainable bamboo forest management and resilience under climate change. By integrating recent findings, we provide a theoretical foundation for advancing research on bamboo-microbe interactions and their applications in sustainable forestry.

## 2 Research hotspots and progress of soil microorganisms with plant

Soil serves as a fundamental environmental component for plant growth, playing a pivotal role in organic matter decomposing, nutrient cycling, and water retention (Ritz et al., 2009; Sağlam et al., 2015; Zhou et al., 2019). As a dynamic medium of plant-microbe interaction, soil harbors a vast and diverse microbial community, with microbial densities up to 10^5^ per gram of soil. These microorganisms are not only crucial for soil ecosystem stability but also serve as sensitive indicators of environmental changes (Xu et al., 2015; Winding et al., 2005). Beyond their role in carbon cycling, soil microbes drive key biogeochemical processes, including nitrogen fixation, phosphorus solubilization, and organic matter decomposition, directly influencing plant health and productivity (Kong et al., 2023). Therefore, the interactions between plants and soil microorganisms represent a crucial focus for advancing the study of plant—microbe relationships.

Microorganisms can be categorized into two types based on their colonization locations: endophytic microbes and those colonizing the surrounding areas of plant, such as phyllospheric and rhizosphere microorganisms. Endophytes were microorganisms that penetrated and invaded plant interiors (Vandenkoornhuyse et al., 2015), and were found in shoots (Martínez-Arias et al., 2021), leaves (Song et al., 2022), buds (Pirttilä et al., 2005), seeds (Wallace, 2023), and roots (Gaiero et al., 2013). Some endophytes promoted host plant growth by secreting hormones like auxin and participating in metabolic regulation, protecting plants from pathogens through autoimmune defense mechanisms (Ali et al., 2012). Phyllospheric microorganisms, also known as epiphytic or foliar microorganisms, resided on plant leaf surfaces (Beattie and Lindow, 1999; Lindow and Brandl, 2003), impacting leaf function, seed quality, fruit development, and host growth homeostasis (Xu et al., 2022). The rhizosphere, a critical plant-microbe interaction area, spaned 0.5–4 mm on the root surface (Kuzyakov and Razavi, 2019). During plant growth, carbon fixed by photosynthesis was released into the rhizosphere as root exudates, including carbohydrates and organic acids, alongside some enzymes (Mommer et al., 2016; Lakshmanan et al., 2014). These root exudates not only provide a carbon source for the soil microbial communities that colonizes the rhizosphere of plants, but also regulate the species, quantity, and distribution of rhizosphere microbes, thus constructing specific rhizosphere microbial community structures (de la Fuente Cantó et al., 2020; Moe, 2013). The rhizosphere microbiome is therefore termed the “Second genome” of plants (Berendsen et al., 2012). Among which, beneficial rhizosphere microorganisms have potential to enhance plant growth by promoting mineral element absorption (Reis and Teixeira, 2015). In summary, microorganisms in different plant tissue parts or growing spaces play irreplaceable roles in plant growth and development.

Plant species and genotypes affect rhizosphere microorganism and endophyte composition. Meanwhile, plant-related microbial community composition and structure are influenced by research methods (Ren et al., 2025; Chen et al., 2022; Dhondge et al., 2022; Li et al., 2020a; Ma and Xiao, 2004; Gao et al., 2004). Two primary methods for studying plant microbial composition: isolating and quantifying microorganisms using various mediums (Krishnapura and Belur, 2016) and sequencing DNA or RNA under culture-free conditions. The former was convenient for studying isolated strains, only a small proportion of natural microorganisms can be cultured in the laboratory (Pace, 1997; Tholozan et al., 1999; Steen et al., 2019), while the latter, a mainstream method, used metagenomics to assess the full DNA data of environmental microorganisms, allowing analysis of complex microbial communities (Hugenholtz and Tyson, 2008; Handelsman, 2004). Metagenomics is widely applied in microorganism research by using length heterogeneity PCR (lh-PCR), PCR-denaturing gradient gel electrophoresis (PCR-DGGE), terminal restriction fragment length polymorphism (T-RFLP) fingerprinting, and sequencing 16S rRNA gene amplicons, to identify culture-independent bacteria (Chen et al., 2022; Dhondge et al., 2022). However, multiple copies of 16S rRNA genes in bacteria introduced detection deviations (Gulati et al., 2011). High-throughput Illumina sequencing is also widely used to study community structure, diversity, and abundance (Yan et al., 2023). New research techniques and analytical models, such as the use of modular toolkits as DNA barcodes for bacterial strains combined with fluorescent proteins, are being developed to track competition between strains in plant tissues and other microbiome members (Ordon et al., 2024). Most plant-associated microbiome research, especially quantitative studies, primarily focuses on relative quantification methods. However, absolute quantification of specific microbial populations with plant tissues or the rhizosphere represents a key advancement in the field, offering more precise and reliable insights into microbial community structure. Absolute quantification methods, such as quantitative PCR (qPCR) or quantitative microbiome profiling (QMP), can overcome many limitations associated with relative quantification, such as primer bias and variations in sample composition. For instance, QMP has been employed to investigate the effects of unbalanced fertilization on soybean rhizosphere microbiome, demonstrating that QMP provides more accurate measurements of specific microbial abundance compared to relative microbiome profiling (RMP) (Wang M. et al., 2024). This approach allows for a more detailed understanding of microbial population dynamics, which is crucial for elucidating plant-microbe interactions.

In addition to these advances in quantification methods, bioinformatics innovations have propelled research into plant-microbe interaction mechanisms, such as microbial metabolism, signal transduction, and genetic regulation. For example, Schäfer et al. (2023) utilized a metagenomic scale model to explore carbon source utilization and the interactions of specific microbial strains on the *Arabidopsis* leaf surface, providing valuable insights into microbial resource allocation and adaption in multispecies environments. The integration of multiple “omics”mics integrationften referred to as “Holo-omics,” is gaining attraction in microbial community research (Odriozola et al., 2024). Holo-omics combines data from genomics, transcriptomics, proteomics, and metabolomics to provide a comprehensive view of biological processes at various levels of organization. This holistic approach enables the identification of dynamic interactions between plants and microorganisms by capturing both microbial and plant responses simultaneously. Furthermore, “Genome-Wide Association Studies” (GWAS) is a powerful method used to identify genetic loci linked to specific traits, such as microbial community composition. By associating genetic variations with microbial phenotypes, GWAS can pinpoint host genes that regulate microbial selectivity and composition in plant tissues. For instance, Zhan et al. (2022) leveraged high-throughput techniques and bioinformatics analyses to identify key microbial communities associated with *Arabidopsis* leaves, and through GWAS and QTLs (Quantitative Trait Locus) mapping, they identified host genes that regulate the microbiota.

Current research on plant-microbe interactions focuses on the relationship between environmental change and microbial community composition and diversity, plant-microbe interaction mechanism, and microbes’ beneficial effects on plants. Studies have examined how diverse elements and temperature changes affects microorganisms (Frey et al., 2023). For instance, long-term nitrogen addition altered the community structure of nitrogen-fixing bacteria in grassland soil without affecting microbial abundance (Frey et al., 2023; Wang et al., 2023). Some studies indicated that microbial responses to warming were weak, with declines in microbial enzyme production, biomass, and other functions based on plant composition analysis (Spinella et al., 2024). Further research by Tao et al. (2024) demonstrated that experimental warming regulated soil priming by altering the active microbial community’s functional structure. Rhizosphere microbiomes are influenced by both soil properties and genes (Xun et al., 2024), emphasizing soil properties’ importance in rhizosphere microorganism composition. Research by Li et al. (2024) showed host plants exhibited strong selectivity for microbes, impacting microbial composition from top to bottom (the effects of plant-driven microbiome assembly), while beneficial microbes played essential roles in plant evolution from bottom to up (microbiome-shaped plant traits). It has been reported that maize-peanut intercropping enriched rhizosphere bacteria associated with secreted iron carriers, increasing iron availability of in intercropping peanut rhizosphere (Wang N. et al., 2024). Beneficial microorganisms promoting plant growth, such as *Bacillus* and *Pseudomonas*, were well studied, mainly in crops like rice, wheat, tomato (Zhou et al., 2021; Dasila et al., 2023; He et al., 2019).

## 3 The diversity and function of microorganisms in bamboos

**FIGURE 1:**
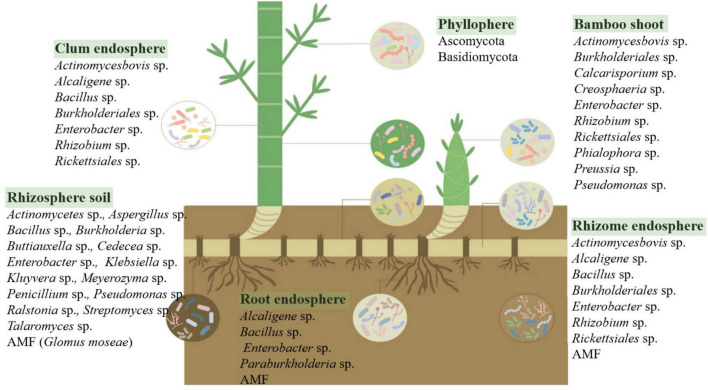
Microbial communities associated with different bamboo niches. It illustrates the distribution of microbial communities across various compartments of the bamboo plant, including the rhizosphere soil, root endosphere, rhizome endosphere, clum endosphere, bamboo shoot, and phyllosphere. The rhizosphere harbors diverse bacteria (*Actinomycetes*, *Bacillus*, *Burkholderia*, *Streptomyces*) and fungi (*Aspergillus*, *Penicillium*, *Glomus mosseae*), playing crucial roles in nutrient cycling and plant growth promotion. The root and rhizome endospheres contain endophytic bacteria (*Alcaligenes*, *Enterobacter*, *Rhizobium*) and arbuscular mycorrhizal fungi (AMF), contributing to nutrient acquisition and stress resilience. The clum endosphere supports structural integrity, while the bamboo shoot microbiome includes plant growth-promoting bacteria (*Pseudomonas*, *Burkholderiales*) and fungi (*Calcarisporium*, *Phialophora*). The phyllosphere is dominated by *Ascomycota* and *Basidiomycota*, which influence leaf surface homeostasis and disease resistance. Understanding these microbial associations provides insights into plant-microbe interactions, disease prevention, and sustainable bamboo management.

Like other terrestrial plants, bamboo comprises two primary parts: the aboveground and underground sections. The aboveground part is exposed to the atmosphere, enabling bamboo to interact closely with various substances and microorganisms. The underground part consists of bamboo roots, and in some cases, bamboo rhizomes, which aid in reproduction. The developed bamboo rhizome system provides sample opportunities for microbial colonization. The microbiome in different bamboo tissues and their surrounding environment exhibits specificity at different growth stages under various environmental conditions (Figure 1, generated by Figdraw), significantly affecting the growth, physiological, and ecological characteristics of bamboo. Therefore, it is valuable to study the microbial diversity and function related to bamboo.

### 3.1 Composition and function of endophere microorganisms

Most land plants are colonized by bacteria (Reinhold-Hurek and Hurek, 2011), fungi (including arbuscular mycorrhizal fungi and other fungi) (Smith and Read, 2008; Vandenkoornhuyse et al., 2007; Streitwolf-Engel et al., 1997), and a few archaea (Sun et al., 2008), with fungi being well studied. Research on the microbial composition in bamboo endophytic tissues mainly focuses on leaves, culms, seeds, and roots. According to previous reports, most known endophytic fungi in bamboo belong to the genus *Ascomycota*, including *Arthrinium, Fusarium, Xylaria*, and *Ascomycota*, and a small variety to Basidiomycota (Wang et al., 2020c). Endophytic bacteria and actinomycetes in bamboos encompass more genera and species, varying in abundance and species across different bamboo varieties and tissues. Studies indicated that endophytes isolated from various bamboo tissues were beneficial for plants growth, exhibiting diverse abilities through functional and genomic analyses, such as promotion plant growth by phosphorus-solubilizing and nitrogen-fixing and inhibiting pathogens. The diversity and function of endophytes in bamboo plants are summarized in Table 1.

**TABLE 1:** Diversity and function of endophytes in bamboos.

Serial number	Techniques for acquiring microbial data	Bamboo species	Tissue	Diversity of endophytes	Predicted/proven microbial function	References
1	High-throughput Illumina sequencing	*Bambusa rigida, Phyllostachys edulis*, and *Pleioblastus amarus*	Leaves	7 phyla, 27 classes, 82 orders, 185 families, 310S genera and 448 species of fungi	/	Yan et al., 2023
2	16S rDNA sequencing	*Phyllostachys edulis*	Rhizome, bamboo culms, bamboo shoots	26 orders of bacteria, including *Actinomycesbovis*, *Rickettsiales*, *Burkholderiales*, *Enterobacteriales*, *Rhizobium*	/	Liu et al., 2017
3	Cultivable bacteria	*Phyllostachys heteroclada*	Shoots and leaves	They belong to Ascomycetes and Basidiomycetes, 6 orders and 14 genera. *Calcarisporium*, *Preussia*, *Creosphaeria* and *Phialophora* have been isolated from bamboos for the first time.	/	Zhou et al., 2017
4	Cultivable bacteria	*Phyllostachys violascens* ‘Prevernalis’	Roots	35 endophytic fungi were isolated	7 strains had the function of cellulose degradation and 9 strains had some inhibition to different pathogenic bacteria	Yang C. et al., 2021
5	Cultivable bacteria	*Phyllostachys edulis*	Seeds	They belong to the phylum Ascomycota and Basidiomycetes, including at least 9 orders and 19 genera, of which four genera are considered to be new records of bamboo fungi in the phylum Basidiomycetes.	Some strains have inhibitory effects on clinical and plant pathogens	Shen et al., 2014
6	Cultivable bacteria and 16S rDNA identification	*Phyllostachys edulis*	Roots	A new strain of *Paraburkholderia* sacchari	Homologous genes encoding the plant growth promoters acetylacetone and butane-2,3-diol biosynthesis have been found in the genome, as well as genes involved in nitrogen fixation and auxin biosynthesis.	Wang K. et al., 2021
7	Cultivable bacteria	*Phyllostachys edulis*	Roots, rhizome, stems, and leaves	A total of 20 phosphate and potassium-solubilizing bacteria, belonging to 14 species of 10 genera, were identified. They were mainly composed of *Alcaligene* sp., *Enterobacter* sp., and *Bacillus* sp., and the *Bacillus* were identified	The function of dissolving phosphorus and potassium	Yuan et al., 2015
8	Cultivable bacteria	*Phyllostachys edulis*	Shoots at different growth stages	A total of 253 strains of endophytic bacteria, RD7-4(*Pseudomonas rhodesiae*), BD24-2(*Burkholderia pyrrocinia*) and TD33-1 (*Pseudomonas edaphica*), were isolated	Phosphate-solubilizing, nitrogen fixation, IAA production. The inoculation could promote the growth of *Arabidopsis thaliana* and *Phyllostachys edulis*.	Zhao et al., 2023
9	Cultivable bacteria	*Phyllostachys edulis*	Bamboo root tissue	The endophytic bacteria CT-B09-2, JL-B06 and WYS-A01-1 were isolated	Promote photosynthesis, chlorophyll content, antioxidant enzyme activity, soluble protein content and soluble sugar content, and reduce malondialdehyde content	Yuan et al., 2018

### 3.2 Composition and function of rhizosphere microorganisms

Studies on the rhizosphere microorganisms of bamboo primarily focus on the quantity of rhizosphere bacteria, fungi, and actinomycetes. These studies often target several widely cultivated bamboo species, such as *Phyllostachys edulis*, *Phyllostachys praecox*, *Fargesia fargesii*, *Fargesia denudata*, and *Phyllostachys heterocycla*.

Under normal growth conditions, the diversity of bacteria and fungi in rhizosphere soil was significantly higher than that in bulk soil (Meng et al., 2015; Qi and Yang, 2006; Li et al., 2020a). High-throughput sequencing results from the rhizosphere soil of various bamboo species indicated that bacteria were the most abundant among rhizosphere microorganisms (Guo et al., 2013). The abundance and diversity of bacterial communities in rhizosphere soils of *Phyllostachys edulis*, *Phyllostachys praecox*, and *Phyllostachys vivax* f. *aureocaulis* surpassed those of fungi (Li et al., 2020b). Consequently, research on bamboo rhizosphere bacteria is more extensive compared to fungi and actinomycetes.

For instance, the average count of bacteria in the rhizosphere soil of *Drepanostachyum luodianense* was 8.32 × 10^7^ CFU/g dry weight, representing the largest number of culturable bacteria in bamboo rhizosphere (Li P. et al., 2016). In contrast, the culturable bacteria in the rhizosphere of *Bashania fangiana* and *F. denudata* were 4.3 × 10^6^ CFU/g dry weight and 1.31 × 10^6^ CFU/g dry weight, respectively (Liu et al., 2008; Qi and Yang, 2006). The bacteria count in the rhizosphere soil of *Phyllostachys edulis* was 7.1 × 10^5^ CFU/g dry weight in Tianmu Mountain and 4.0 × 10^5^ CFU/g dry weight in Jinyun Mountain, both lower than those in *Bashania fangiana* and *Fargesia denudata* (Li et al., 2008).

However, fewer studies focus on the count of fungi in bamboo rhizosphere soil, mainly addressing *Fargesia denudata* and *Phyllostachys edulis*. Qi and Yang (2006) estimated that the average count of fungi in the rhizosphere soil of *Fargesia denudata* was 1.02 × 10^5^ CFU/g dry weight. The fungi count in the rhizosphere soil of *Drepanostachyum luodianense* was 9.19 × 10^5^ CFU/g dry weight, comprising 34 species across 12 genera (Li P. et al., 2016; Chen M. et al., 2021).

The count of actinomycetes in the rhizosphere soil varies significantly between different bamboo species and the same bamboo species in different areas. For example, the actinomycetes count in the rhizosphere soil of *Phyllostachys edulis* in Tianmu Mountain and Jinyun Mountain were 3.0 × 10^5^ CFU/g dry weight and 3.3 × 10^3^ CFU/g dry weight, respectively (Li et al., 2008). A total of 20 actinomycetes were isolated from the rhizosphere soil of *Phyllostachys heterocycla*, belonging to 15 species within one genus, with a count of 2.32 × 10^6^ CFU/g dry weight (Qi and Yang, 2006; Chen M. et al., 2021).

The results showed that the diversity of bacteria, fungi and actinomycetes in the rhizosphere soil of bamboo plants had a certain correlation with bamboo species, this may be related to the composition of bamboo root exudates, the growth environment of bamboo species and the soil environment. Bacteria are more abundant than fungi, probably because bamboo plants produce more simple organic compounds in root exudates, and bacteria utilize simple organic compounds more efficiently and compete with fungi for complex compounds, which may contribute to the abundance of bacteria, allowing more bacteria to be recruited (Wang and Kuzyakov, 2024). The number of culturable microorganisms in the rhizosphere of bamboo plants is much less than the total number, which may be due to the fact that most microorganisms cannot be cultured in laboratory conditions away from the natural environment.

Several rhizosphere microorganisms isolated and cultured from bamboo have demongstrated positive effects on plant growth in experimental studies. These benificial rhizosphere bacteria, collectively known as plant growth-promoting rhizobacteria (PGPR) (Kloepper, 1981), play diverse roles in enhancing bamboo growth. They contribute by increasing biomass accumulation, facilitating nutrient uptake, and suppressing pathogenic infections. Various PGPR species have been identified in the bamboo rhizosphere, each exhibiting distinct mechanisms such as nitrogen fixation, phosphate solubilization, and phytohormone production, as summarized in Table 2.

**TABLE 2:** Function and function of rhizosphere microorganisms in bamboo plants.

Serial number	Bamboo species	Classification of microbial strains	Species of rhizosphere microorganisms	Microbial function	The effects on plants and their surroundings	References
1	*Phyllostachys edulis*	Bacteria	*Burkholderia* sp. and *Raoultella* sp.	Promote the growth of plants	The biomass of *P. edulis* and diversity of soil microbial community were significantly increased, and soil nutrient content and enzyme activity were improved	Li et al., 2023
2	*Phyllostachys edulis*	Fungi	*Talaromyces aurantiacus*, *Aspergillus neiger*	Phosphate solubilization -solubilizing	*Talaromyces aurantiacus* enhanced the supply of available phosphorus and significantly increased the biomass of *Phyllostachys edulis*	Yang D. et al., 2021
3	*Phyllostachys sulphurea*, *Phyllostachys bambusoides, Sinobambusa tootsik, Sasa auricoma*	Bacteria and fungi	52 strains of phosphate solubilization bacteria were identified to belong to 10 genera of bacteria and 4 genera of fungi	The *Bacillus*, *Kluyvera*, *Buttiauxella*, *Meyerozyma* and *Penicillium* have the ability to digest both organophosphorus and inorganic phosphorus	/	Xing et al., 2021
4	*Phyllostachys edulis*	Bacteria	*Ralstonia* sp., *Klebsiella* sp. *and Enterobacter* sp.	Dephosphorization	Significantly increase the root activity and total chlorophyll content of *Phyllostachys edulis* leaves and soil microbial association, accelerate biomass accumulation	Liu Y. et al., 2023; Li et al., 2023
5	*Phyllostachys edulis*	Bacteria	*Bacillus polymyxa, Bacillus licheniformis*	Nitrogen fixation	Improve the survival rate of bamboo seedlings, significantly improve the fresh weight of bamboo roots. The above-ground, underground and total dry weight of tissue-cultured *Dendrocalamus latiflorus* seedlings were increased	Gu and Wu, 1999
6	Unknown species of bamboo	Actinomycetes	*Actinomycetes Streptomyces* BS-16	Resistance to pathogens	It has significant antibacterial activity against *Staphylococcus aureus* (19 mm), *Bacillus subtilis* (12 mm) and *Streptococcus pyogenes* (10 mm)	Sriragavi et al., 2023

In addition to PGPR, arbuscular mycorrhizal fungi (AMF), have been found to establish symbiotic associations with bamboo roots. AMF, which colonized the roots of approximately 80% of land plants (Vierheilig et al., 1998), play a crucial role in improving nutrient acquisition and stress tolerance. Studies have indicated that AMF inoculation enhanced bamboo growth by increasing phosphorus uptake, improving root system development, and potentially boosting resistance to abiotic stresses (Verma and Arya, 1998). Despite limited research on AMF-bamboo interactions, available findings highlight their significant contribution to bamboo health and productivity.

Although some microorganism strains are not originally isolated from the rhizosphere of bamboos, the growth of bamboos can be significantly improved by introducing these strains into the rhizosphere through liquid medium. This method has been shown to increase bamboo biomass and root length. Details of these reported strains and their effects are summarized in Table 3.

**TABLE 3:** Promotion of beneficial strains on bamboo growth.

Serial number	Method of inoculation	Classification of strains	Species of rhizosphere microorganisms	Microbial function	Effect on the growth of bamboos	References
1	Drip the bacterial suspension	Bacteria	*Bacillus polymyxa*, *Bacillus licheniformis*, *Klebsiella pneumoniae*	Nitrogen fixation	Different strains from *Bacillus licheniformis* Chester (14#) and *Klebsiella pneumoniae* (Schroeter) Trevisan (7#) significantly increased the biomass of *Dendrocalamus latiflorus* tissue culture plantlets	Gu et al., 2001
2	Smear the bacterial suspension	Bacteria	*Bacillus amyloliquefaciens*	Promote the growth of plants	Significantly increased root length, rooting number and first-order lateral root length of *Dendrocalamus brandisii*, *Dendrocalamus birmanicus, Dendrocalamus Giganteus* and *Bambusa Lapidea*	Yin et al., 2011
3	Add the fungal inoculum	Fungi	AMF	Promote the growth of plants	The above-ground plant height, dry weight and phosphorus absorption capacity of the whole plant of *Bambusa bambos* and *Dendrocalamus strictus* were increased	Muthukumar and Udaiyan, 2006
4	Irrigate the bacterial suspension	Bacteria	*Bacillus amyloliquefaciens* B01-2 and *Bacillus subtilis* b23-1 were mixed	Prevent and cure pathogenic bacteria and promote growth	The activities of defense enzymes such as Peroxidase (POD), Polyphenol oxidase (PPO) and Phenylalanine ammonia-lyase (PAL) in hybrid bamboos were increased after treatment with the fermentation broth of single and mixed strains, reduce the decomposition of chlorophyll and the production of malondialdehyde (MDA) in bamboo leaves, improve the plant immunity	Li et al., 2018
5	Microbe inoculum injection	Bacteria and Fungi	Mix of 15 PSM strains (including *Burkholderia* sp., *Pseudomonas* sp., *Cedecea* sp., *Enterobacter* sp., *Burkholderia* sp., *Enterobacter* sp., *Bacillus* sp., *Raoultella* sp., *Enterobacter* sp., *Burkholderia* sp., *Pseudomonas* sp., *Bacillus* sp., *Pseudomonas* sp., *Meyerozyma* sp., *Penicillium* sp.), and AMF *(Glomus moseae)*	Enhance P activation and uptake by plants	The utilization of PSMs, especially in combination with AMF, proves to be an effective strategy for enhancing *P. edulis* seedlings growth during their second-year growth, particularly in P-deficient soil.	Xing et al., 2024
6	Microbe inoculum injection	Fungi	AMF	Promote the growth of plants	The *Dendrocalamus asper* significantly increased the above-ground phosphorus concentration, promoted root colonization and spore production	Verma and Arya, 1998

### 3.3 Composition and function of microorganisms in phyllosphere

The systematic and thorough study of plant microecology is conducive to understanding the relationship between microorganisms and plants. Phyllospheric microorganisms are an important part of plant microecology. It is significant to enrich microbial sources and develop new applications in the control of plant diseases by intensive research. Compared to rhizosphere microbes, research on the composition and diversity of the microbes in bamboo leaves is less extensive. Using ITS1 amplification and metagenomic sequencing, Kang et al. (2022) analyzed the diversity and function of phyllosphere fungi in three main bamboo species preferred by panda: *Arundinaria spanostachya*, *Yushania lineolata*, and *Fargesia Ferax*. The results showed that the predominant fungi were Ascomycota and Basidiomycota, and their relative abundance did not significantly differ among the three bamboo species.

As the sole food source for giant panda, bamboo is high in cellulose. However, giant pandas lack the genome-encoding enzyme necessary to digest cellulose (Hu et al., 2017), suggesting that microbial degradation plays a significant role in bamboo digestion (Li et al., 2010). Some bacterial phyla such as Proteobacteria, Acidobacteria, and Bacteroidetes in bamboo phyllospheres are dominant (Kang et al., 2022). These microbes may contribute to the breakdown of complex plant materials and improve digestion. Additionally, these microbiota play important biological roles in promoting plants growth and development, such as regulating root growth, promoting nutrient absorption, balancing plant hormones, and preventing disease invasion (Xu et al., 2018). An imbalance in the panda’s gut flora can lead to gastrointestinal diseases, which are the most common cause of death in pandas (Tun et al., 2014). As an important food source for giant pandas, bamboo leaves have different nutrient and microbial compositions as compared with bamboo shoots, bamboo stems, and branches (Wei et al., 2015; Wu et al., 2017; Long et al., 2021). Thus, the microbes on the bamboo leaves may influence the composition and changes in the panda’s gut flora after consuming bamboo leaves. Therefore, studying the composition and abundance of microbes in the phyllosphere of bamboo plants, especially those species consumed by giant panda, is crucial for maintaining the normal growth and population stability of giant panda. Moreover, further research is needed to identify microbial communities in bamboo foliage that benefit both giant panda digestion and bamboo plant growth.

## 4 Factors affecting the microbial composition of bamboos

**FIGURE 2:**
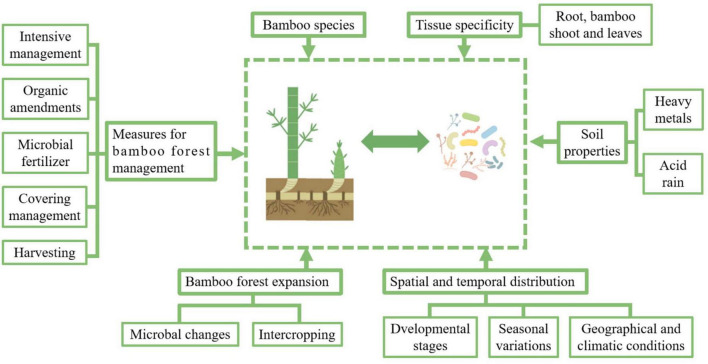
Key factors influencing the bamboo-associated microbiome. This figure illustrates the diverse factors shaping the composition and dynamics of the bamboo-associated microbiome. The microbiome is influenced by bamboo species and tissue specificity, with distinct microbial communities colonizing roots, shoots, and leaves. Soil properties, including heavy metal contamination and acid rain, impose selective pressures that alter microbial diversity and function. The spatial and temporal distribution of microbes varies with developmental stages, seasonal changes, and geographical and climatic conditions, reflecting dynamic shifts in plant-microbe interactions. The expansion of bamboo forests, whether through natural spread or intercropping, affects microbial composition by modifying soil environments and plant-microbe relationships. Additionally, management practices, such as intensive cultivation, organic amendments, microbial fertilizers, covering techniques, and harvesting methods, play a crucial role in structuring microbial communities. These practices can enhance soil health, promote beneficial microbes, and improve bamboo resilience. Understanding these interconnected factors provides insights into microbiome regulation and offers strategies for optimizing bamboo forest management to support sustainable growth and ecosystem stability.

According to previous researches, the main factors affecting the microbial composition of bamboo plants include bamboo species, tissue specificity, spatial and temporal distribution, soil properties, management measures, and the expansion of bamboo forests (Figure 2, generated by Figdraw).

### 4.1 Bamboo species

Plants recruit soil microorganisms to colonize the rhizosphere through root exudates, resulting in variations in rhizosphere microbe communities among different plants (Xia et al., 2023; Dawson et al., 2017). Similar to crop studies, the bacterial communities at the phylum level with high relative abundance in the rhizosphere of rice are *Proteobacteria*, *Actinobacteria*, *Gemmatimonadetes*, and *Acidobacteria* (Zhang Z. et al., 2015), while those in wheat are *Proteobacteria*, *Bacteroidetes*, Acidophilus and Chloroflexi (Wang et al., 2019), demonstrating that there are different from each other in the bacterial communities.

High-throughput sequencing studies on the rhizosphere microbial composition of common bamboo species in the genus *Phyllostachys* revealed differences in dominant microbial populations (Li et al., 2020a; Fu et al., 2022). The rhizosphere soils of several bamboo species, including *Phyllostachys edulis*, *Phyllostachys glauca*, *Phyllostachys vivax* f. *aureocaulis*, and *Phyllostachys iridescens*, contained dominant bacteria such as Proteobacteria, Chloroflexi, Actinobacteria, and Acidobacteria (Li et al., 2020b; Fu et al., 2022). Although these dominant bacterial phyla are consistent across species, their relative abundance varies. For instance, the relative abundance of the four dominant bacterial phyla, including Proteobacteria, Chloroflexi, Actinobacteria, and Acidobacteria, in the rhizosphere of *Phyllostachys edulis* is similar, in contrast, Proteobacteria and Acidobacteria are most abundant in *Phyllostachys praecox* and *Phyllostachys vivax* f. *aureocaulis*, respectively. The highest relative abundance of Actinomycetes is found in the rhizosphere soils of *Phyllostachys glauca* and *Phyllostachys iridescens*.

At the class level, Alphaproteobacteria and Acidobacteria dominate the rhizosphere soils of *Phyllostachys edulis*, *Phyllostachys praecox*, and *Phyllostachys vivax* f. *aureocaulis*, whereas Actinobacteria dominate *Phyllostachys glauca* and *Phyllostachys iridescins*, indicating significant differences in relative abundance among microbial classes in rhizosphere soils of different bamboo species. For example, the relative abundances of Thermoleophilia in the rhizosphere soils of *Phyllostachys edulis*, *Phyllostachys glauca*, and *Phyllostachys iridescens* are significantly different, with the highest abundance in *Phyllostachys glauca* and the lowest in *Phyllostachys edulis*. Similarly, the relative abundances of Gammaproteobacteria, Bacteroidia and Bacilli exhibit extremely significant difference, with Gammaproteobacteria being most abundant in *Phyllostachys edulis* and least abundant in *Phyllostachys glauca*. At the order level, Rhizobiales is dominant in the rhizosphere soil of *Phyllostachys edulis*, while both Rhizobiales and Acidobacteriales dominate in the rhizosphere soils of *Phyllostachys praecox* and *Phyllostachys vivax* f. aureocaulis. Both *Phyllostachys edulis* and *Phyllostachys praecox* need to absorb large amounts of water during their growth, otherwise they may wilt. Previous study reported that when exposed to drought stress, rhizobia, as beneficial bacteria, were enriched in the rhizosphere to improve drought resistance by uptaking nutrients and synthesizing plant hormones (Chen et al., 2024), inferring that may be the reason why Rhizobiales are the dominant class in the rhizosphere soil of these two bamboo species. Rhizosphere fungal communities of *Phyllostachys edulis*, *Phyllostachys glauca*, and *Phyllostachys iridescens* are primarily composed of Ascomycota and Basidiomycota, with no significant differences at the phyla level. However, at the class level, Sordariomycetes is more abundant in *Phyllostachys glauca*, Leotiomycetes in *Phyllostachys iridescens*, and Eurotiomycetes in *Phyllostachys edulis*. Additionally, unique differences at the order level were detected, such as the presence of the unique fungi family Helotiaceae in the rhizosphere soil of *Phyllostachys edulis* (Fu et al., 2022).

Moreover, the composition of endophytic fungi and the abundance of soil microorganisms vary among bamboo species. For instance, *Pleioblastus amarus* had the highest abundance of endophytic fungi, followed by *Phyllostachys edulis* (Yan et al., 2023). The abundance of soil bacteria in the three bamboo forests followed the order *Phyllostachys edulis* > *Bambusa pervariabilis* × *Dendrocalamopsis daii* > *Bambusa emeiensis* (Xu et al., 2022).

### 4.2 Tissue specificity

The microbial community structure in different tissues of bamboo exhibits significant variation, with distinct dominant phyla and diversity levels across tissues such as leaves, roots, rhizosphere soil, and non-rhizosphere soil. Comparative analyses of these microbial communities reveal marked differences in both bacterial and fungal populations. By studying the changes of microbial communities in different tissues under various growth conditions, strains actively responsive to stress can be screened and made into microbial fertilizers to regulate the growth of bamboo plants, improving the ability to cope with environmental stress.

#### 4.2.1 Microbial community structure in different tissues

Studies using principal coordinate analysis (PCoA) and hierarchical cluster analysis (HCA) based on the Bray-Curtis difference matrix demonstrated significant differences in bacterial diversity and composition between the surface of leaves and roots in various bamboo species. These differences were apparent in β diversity but not in α diversity, indicating variations in community composition rather than richness (Zheng and Lin, 2020).

The bacterial communities in the rhizosphere soil and endophytic tissues of *Phyllostachys edulis* differ significantly from those in non-rhizosphere soil, rhizome soil, and root endophytic tissues (Yuan et al., 2021). For example, endophytic bacterial communities in bamboo shoots and culms showed distinct compositions, emphasizing tissue-specific microbial associations (Liu et al., 2017).

Recent high-throughput sequencing analyses of *Phyllostachys edulis* revealed that Proteobacteria dominated in bamboo shoot and rhizome samples, while Acidobacteria were prevalent in rhizosphere and forest soil samples. The main genera in rhizome samples included *Acidothermus*, *Bradyrhizobium*, and *Acidobacterium*, whereas soil samples were dominated by *Acidothermus* and *Acidobacterium* (Yuan et al., 2023). This indicates a clear distinction in microbial communities between soil-associated and plant tissue-associated niches.

#### 4.2.2 Differences in dominant microbial phyla

The dominant phyla in various tissues of bamboo also show significant variation. For instance, the abundance of Proteobacteria and Actinomycetes was notably lower in the root endosphere of *Fargesia spathacea* compared to the rhizosphere and root zone. Conversely, the dominant fungi, Ascomycetes and Basidiomycetes, exhibited higher abundances in the root endosphere compared to the rhizosphere and root zone (Zhang et al., 2023). This contrast highlights the influence of tissue-specific environments on microbial community structure.

### 4.3 Spatial and temporal distribution

Microbes play crucial roles in plant growth and development, and the feedback regulation by plants can significantly alter microbial community structures and functions. Consequently, microbial communities in bamboo plants exhibit significant variation over different spatial and temporal scales. These changes are influenced by factors such as the developmental stages of bamboo, seasonal variations, and geographical conditions.

#### 4.3.1 Developmental stages of bamboo

The composition of microbial communities in the rhizosphere of bamboo changes with the age of the bamboo. For instance, in *Fargesia denudata*, the total number of bacteria, fungi, and other microorganisms increased with bamboo age up to the 5-year-old and then decreased from 7- to 13-year-old (Qi and Yang, 2006). Similarly, in *Phyllostachys edulis*, the bacteria count was significantly higher in the rhizosphere soil of young bamboo compared to mature and old bamboo, while the fungi count was the highest in the rhizosphere soil of mature bamboo (Meng et al., 2015). However, the count of endophyte in bamboo culms increased with bamboo growth (Liu et al., 2017).

Changes in soil microbial communities are also observed with increasing forest age. In a study of *Phyllostachys edulis* forests at 19, 37, and 64 years, the relative abundances of Alphaproteobacteria, Gammaproteobacteria and Bacteroidetes increased significantly with forest age, while the relative abundances of Acidobacteria and Verrucomicrobia decreased (Yang Y. et al., 2021). Using phospholipid fatty acid (PLFA) analysis, it was found that the microbial community structure of II degree bamboo in rhizosphere soil differed significantly different from that of other ages (Zhang W. et al., 2015). Both flowering and non-flowering *Phyllostachys edulis* rhizosphere soils contain Proteobacteria and Acidobacteria strains, but the ratio of Firmicutes in flowering *Phyllostachys edulis* rhizosphere is slightly higher than in non-flowering *Phyllostachys edulis* rhizosphere, while the counts of Rokubacteria, Latescibacteria and Chlorofexi decrease significantly. This may be due to the stress resistance, different nutrient condition adaptions and plant immune responses (Yuan et al., 2021). At the genus level, the abundance of denitrifying bacteria *Stenotrophomonas* in the rhizosphere soil of non-flowering *Phyllostachys edulis* is significantly higher than in flowering *Phyllostachys edulis*; after flowering, the abundance of *Flavobacterium*, which can decompose organic matter, is much lower in the rhizosphere soil than in non-flowering *Phyllostachys edulis*, while the abundance of *Bacillus* responding to nitrogen change is over seven times higher than in non-flowering *Phyllostachys edulis*.

#### 4.3.2 Seasonal variations

Seasonal changes, closely related to temperature, have a profound effect on the microbial communities in bamboo rhizospheres. Tao et al. (2024) demonstrated that simulated warming could alter the functional structure of the active microbial community to regulate the soil excitation effect. *Cephalostachyum pingbianense*, an endemic bamboo species found in southern Yunnan, is currently the only bamboo species known to produce shoots year-round. Li et al. (2022) showed that there was a positive correlation between the shoot yield of *Cephalostachyum pingbianense* and microbial communities involved in carbon and nitrogen cycling. Although seasonal variation has no significant effect on the α-diversity of rhizobacteria in *Cephalostachyum pingbianense*, it strongly impacts the community structure of bacteria and fungi, as well as the total abundance of fungi. During the four seasons, bacteria genera such as *Acidotherus* sp., *Roseiarcus* sp., and *Bradyrhizobium* sp., and fungi genera such as *Saitozyma* sp. *Mortierella* sp., and *Trichoderma* sp. are dominant in rhizosphere soil of *Cephalostachyum pingbianense*.

In *Phyllostachys reticulata*, unique bacterial strains are isolated in each season. The bacterial richness index was highest in September, and diversity indices (Simpson, Shannon-Wiener, and evenness) increased from April to September (Yin et al., 2016). Similarly, in *Phyllostachys edulis* forests, the Shannon diversity of rhizosphere microbiome varies significantly between on-year and off-year periods during defoliation and growing seasons (Wang Y. et al., 2024). Seasonal studies of various bamboo species, such as *Bambusa emeiensis* and *Bambusa pervariabilis* × *Dendrocalamopsis daii*, showed that bacteria and actinomycetes were most abundant in summer and least in winter, while fungi peaked in summer and increased slightly in winter (Xu et al., 2015).

#### 4.3.3 Geographical and climatic conditions

Geographical and climatic conditions also affect the rhizosphere microbial composition of bamboo. Comparing *P. edulis* rhizosphere microbial communities in Tianmu Mountain and Jinyun Mountain, the diversity and evenness were higher in Tianmu Mountain. Unique bacterial strains such as *Deinococcus* were found in Tianmu Mountain, while *Microbacterium* was exclusive to Jinyun Mountain. Tianmu Mountain has a subtropical climate with a more humid climate and more solar radiation than Jinyun mountain area, which may result in the various microbial community composition. Subdominant genera also differed, with *Streptomyces* in Tianmu Mountain and *Acinetobacter* in Jinyun Mountain (Li et al., 2008).

Slope direction and altitude significantly influence soil microorganisms in bamboo. In *Dendrocalamus giganteus*, bacterial diversity, fungal diversity, and bacterial abundance were higher on sunny slopes of semi-natural forests than in other plots (Liu et al., 2020). Microbial community studies at different altitudes (600–1,800 m) in *Phyllostachys edulis* showed that Acidobacteria and Proteobacteria were dominant across altitudes, but their relative abundances varied. The microbial diversity was highest at mid-altitudes (1,000 –1,200 m) (Lin et al., 2015).

### 4.4 Soil properties

The distribution of bamboo forests across diverse geographical and climatic regions results in a variety of soil types and properties. This diversity influences the composition and structure of soil microorganisms and endophytic microbial communities in bamboo. Conversely, soil microorganisms can alter the physical and chemical properties of their surrounding soil, highlighting a bidirectional relationship between soil properties and microorganisms (Philippot et al., 2024).

#### 4.4.1 Influence of soil properties on microbial communities

The rhizobacteria community diversity and structure in *Cephalostachyum pingbianense* are mainly affected by soil temperature, water and organic matter, while the fungal community structure is also influenced by soil temperature and water, with available phosphorus in soil having the greatest effect on fungi diversity (Li et al., 2022). It may be caused by the decrease of available phosphorus in soil, which promotes the symbiosis between plants and mycorrhizal fungi, thereby increasing the phosphorus uptake by plants (Shi et al., 2021), inferring that the enrichment of mycorrhizal fungi may improve the fungal community diversity. In *Phyllostachys praecox*, soil pH, total organic carbon (TOC), total nitrogen (TN), total phosphorus (TP), available potassium (AK), and TOC/TP ratio were highly correlated with the bacterial community composition of rhizosphere soil and root endophytes (Zhang et al., 2022a). Intensive management of *Phyllostachys praecox* also showed significant correlations between available nitrogen (AN), available phosphorus (AP), AK, TOC, and the composition of endophytic bacterial communities (Zhang et al., 2019a). Specifically, TOC, AN, AP, and pH are closely related to bacterial community composition, while AK and pH significantly influence fungal community composition. In conclusion, the high correlation between microbes and soil properties provides the possibility to modify bamboo bacterial community composition of rhizosphere soil and root endophytes by regulating soil properties.

#### 4.4.2 Impact of heavy metals

High levels of heavy metals in soil can significantly alter the composition of rhizosphere microorganisms. There is a negative correlation between the Shannon index for bacteria and fungi and the presence of chromium (Cr) in the soil. The community composition of bacteria and fungi in the rhizosphere soil of *Phyllostachys praecox* is significantly correlated with soil Cr, pH, TOC, AP and AN (Zhang et al., 2020). In cases of heavy metal pollution, such as with total chromium (TCR) and extractable chromium (ACR), there is a negative correlation with the soil bacterial α-diversity indices (Chao1 and Shannon). Interestingly, this negative correlation is not observed for fungi, indicating that fungi are more resistant to chromium contamination (Zhang et al., 2021).

#### 4.4.3 Effects of acid rain

Changes in external conditions, such as acid rain, can affect soil microbial composition by changing soil physical and chemical properties, notably soil pH. Under simulated acid rain conditions with a pH 2.5, the diversity indices of fungal community in *Phyllostachys edulis* soil increases, while the diversity and abundance of soil bacteria decrease (Wang et al., 2020a). Simulated acid rain also affects the structure of soil fungal community, changing the relative abundance of genera such as *Bifiguratu*, *Geminibasidium*, *Purpureocillium*, and *Oidiodendron* (Wang et al., 2020b).

### 4.5 Measures for bamboo forest management

Effective bamboo forest management involves various practices aimed at increasing forest yield per unit area. These measures primarily include intensive management and covering management. Each approach influences the composition and structure of microbial communities in different ways.

#### 4.5.1 Intensive Management

Intensive forest management includes practices such as fertilization, removal of understory vegetation, and deep ploughing (Xu et al., 2008). For monopodial bamboo, which absorbs nutrients from the soil through an extensive underground system, these practices can significantly impact the microbial communities in the rhizosphere. For instance, the count of bacteria in rhizosphere soil decreased with intensive management post-deep ploughing and fertilization in *Phyllostachys edulis* plantations. However, there is no significant difference among treatments of deep plowing with biennial fertilization, quadrennial fertilization, and no-tillage with no fertilization. Intensive management also significantly reduces soil fungi populations, and increases the bacterial-to-fungal ratio (Ni et al., 2021). There was also a study revealed that intensive management measures like deep ploughing, fertilization, and organic mulching changed soil properties in *Phyllostachys edulis* forests, reducing Shannon index of microbial communities in both soil and rhizome, though they had no significant effect on community composition (Zheng et al., 2022).

Additionally, duration of intensive management also affects the associated microbiome of bamboos. Over 5, 10, 15, and 20 years, intensive management increases the relative abundance of Firmicutes and Bacteroidetes and decreases Proteobacteria. However, relative abundance trends vary over these periods, with significant differences in some phyla. For instance, Acidobacteriota abundance in *Phyllostachys praecox* plantation roots under 15 years of intensive management is not significantly different from normal conditions, whereas it increases or decreases significantly at other times (Zhang et al., 2019a). Concomitantly, intensive management increases the relative abundance of Actinobacteria and Crenarchaeota and decreases Acidobacteria in bacterial community of *Phyllostachys edulis* forest soil (Li Q. et al., 2016). It also alters fungal communities, increasing Basidiomycota abundances and Mortierellomycota while decreasing Ascomycota and R*ozellomycota* (Zhang et al., 2022b).

Furthermore, intensive management can facilitate pathogen invasion. For instance, intensive management conferred to a decrease in the soil microbial diversity of *Phyllostachys edulis* during pathogenic *Escherichia coli* invasion, indicating that intensive management benefits pathogen invasion but is detrimental to maintain soil microbial diversity (Qin et al., 2023). According to Niu et al. (2017), long-term extensive management resulted in a gradual decrease in soil microbial richness and diversity in *Phyllostachys edulis* forest. Abandoned farmland also affected soil microbial communities in bamboo forest, with soil bacterial community indices (e.g., Shannon, Simpson, and Chao1) increasing and then decreasing over time (Jin et al., 2023).

#### 4.5.2 Organic amendments and microbial fertilizer application

Organic amendments significantly increase the relative abundance of Proteobacteria and decrease Acidobacteria, Bacteroidota, and Verrucomicrobia in rhizosphere and endophytic microbial communities of *Phyllostachys praecox* plantations (Zhang et al., 2022a). In contrast to untreated *P. praecox* roots, organic amendments increase endophytic Proteobacteria and Acidobacteria while decreasing Actinobacteria and Firmicutes. Proteobacteria and Acidobacteria dominate the rhizosphere microbiome, suggesting they could be indicators of root-related microbiome response to organic amendments. Lack of organic amendments increases the complexity of the microbial network of root endophytes but decreases that of rhizosphere soil. Combining organic fertilizer with *Bacillus amyloliquefaciens* bio-fertilizer or *Bacillus mucilaginosus* Krassilnikov bio-fertilizer significantly affects soil bacterial species’ relative abundance (Li et al., 2023). Organic amendments and bio-fertilizers significantly improve soil microflora in various bamboo species (Singh et al., 2020).

#### 4.5.3 Covering management

Covering management regimes like mulching is generally used in bamboo forests, especially in *Phyllostachys praecox*, to enhance bamboo shoot production, affecting soil microbial composition (Zhao et al., 2015). Mulching initially increases soil bacteria and actinomycetes, followed by a decrease, while fungi populations significantly increase over time (Zhai et al., 2017). Furtherly, Mulching affects the population of ammoniacal bacteria but not nitrogen-fixing bacteria (Luo et al., 2016). Addition, mulching time and materials significantly impact bacterial community composition and diversity. For instance, a 4-month covering treatment with wheat straw and chicken manure reduces α-diversity of soil bacterial community in *Phyllostachys praecox* forest (Wu et al., 2023). For *Phyllostachys edulis*, mulching alters Chao1 and Simpson indices (Sun et al., 2023a).

#### 4.5.4 Harvesting

Rational harvesting and management practices, such as strip cutting, can improve productivity and ecological balance in bamboo forests (Zhao et al., 2016). Strip cutting creates forest gaps and influences the bamboo forest ecosystem (Amir and Duke, 2019). Cutting width significantly affects soil bacterial phyla abundance. A 3-m cutting width results in the highest bacterial community abundance and uniform species distribution (Wang S. et al., 2021). Increasing cutting width decreases bacterial community abundance and species evenness while increasing Proteobacteria and decreasing Acidobacterium and Campylobacter (Wang S. et al., 2021; Wang et al., 2022).

### 4.6 Effects of bamboo forest expansion on soil microorganisms

The expansion of bamboo forests, particularly *Phyllostachys edulis*, can significantly affect soil microorganisms. The rhizome system of scattered bamboos enables lateral growth, allowing them to invade other natural and secondary forests, thereby altering the rhizosphere and soil microbial composition of native plants.

#### 4.6.1 Impact on rhizosphere soil microorganisms

For instance, the invasion of *Phyllostachys edulis* increases the abundance and diversity of denitrifying bacteria in the rhizosphere soil of *Robinia pseudoacacia*, including *Shewanella*, *Chitinophaga*, and *Achromobacter*. The higher the level of invasion, the more denitrifying bacteria are present in the forest soil. This invasion changes the nitrogen cycle in the settled habitat by altering the population of denitrifying bacteria (Cao et al., 2020). *Shewanella* species, often isolated from water, are capable of bioremediation of metal contaminants and can naturally break down harmful compounds (Lemaire et al., 2020). Consequently, the content of harmful compounds in the soil may be reduced post-invasion, indirectly enhancing the stress resistance of native tree species.

#### 4.6.2 Changes in soil microbial diversity and biomass

The transitioning from broad-leaved forests to *Phyllostachys edulis* forests results in a significant reduction in soil bacterial richness and diversity, with minimal impact on bacterial evenness. In contrast, fungal diversity shows a marked increase under *Phyllostachys edulis* dominance. Specifically, the soil microbial richness index is significantly lower in *Phyllostachys edulis* forests compared to both bamboo-broad-leaved and broad-leaved forest ecosystems. Additionally, microbial diversity indices, including the ACE index and Shannon index, are notably reduced in *Phyllostachys edulis* and mixed forests relative to broad-leaved forests (Ma et al., 2022). The significant increase in fungal diversity when *Phyllostachys edulis* was used as the dominant position in invasion may be due to the increased secretion of complex components of root exudates of other plants affected by *Phyllostachys edulis*, attracting fungi for utilization (Wang and Kuzyakov, 2024). In terms of microbial biomass, *Phyllostachys edulis* invasion significantly enhances soil microbial biomass nitrogen, fungal ACE diversity, fungal biomass, and bacterial diversity. This expansion also alters the microbial community structure by reducing the Gram-positive to Gram-negative bacteria ratio, alongside a decrease in the biomass of Gram-positive bacteria (Luo et al., 2024). Furthermore, *Phyllostachys edulis* invasion increases the operational taxonomic units (OTUs) of fungi in both coniferous and broad-leaved forest soils, indicating a higher fungal species diversity in these ecosystems. However, the invasion slightly decreases bacterial OTUs and the ACE index (a measure of microbial species richness) in broad-leaved forest soils, suggesting a shift toward a more specialized microbial community under bamboo dominance (Wu et al., 2024). These results highlight the complex, multifaceted influence of bamboo invasion on soil microbial dynamics, which may have important implications for forest management and ecosystem function.

#### 4.6.3 Relative abundance of soil fungi and bacteria

The relative abundance of fungal families Hyaloscyphaceae (Ascomycota), Clavulinaceae, Hydnangiaceae, Inocybaceae, Russulaceae, Sebacinaceae, Thelephoraceae (Basidiomycota) were higher in broad-leaved forest soil compared to *Phyllostachys edulis* forest soil. Conversely, the relative abundance of families Herpotrichiellaceae (Ascomycota), Entolomataceae, Hydnodontaceae, Hygrophoraceae were more abundant in *Phyllostachys edulis* forest soil, indicating significant changes in the fungal community post-invasion (Chen Z. et al., 2021). There were also studies showing that that the expansion of bamboo forests can significantly influence the composition and diversity of soil bacterial communities (Tian et al., 2020). This impact is primarily attributed to changes in soil properties such as pH, organic matter content, and nutrient availability, all of which are affected by bamboo growth and litter input. For instance, Zhang (2023) investigated soil bacterial communities in a cedar plantation invaded by Moso bamboo and found significant shifts in bacterial composition, highlighting alterations in soil microbial dynamics.

#### 4.6.4 Effects on arbuscular mycorrhizal fungi

*Phyllostachys edulis* invasion alters the arbuscular mycorrhizal fungi (AMF) community structure in both broad-leaved and coniferous forests. The AMF richness (Chao1) is significantly higher in mixed bamboo-broadleaved forests than in pure bamboo forests. The relative abundance of *Acaulosporaceae* and *Archaeosporaceae* is lower in bamboo forest soil compared to broad-leaved forest soil (Qin et al., 2017). The AMF community composition varies significantly across *Cedrus deodara*, *Phyllostachys edulis*-*Cedrus deodara* and *Phyllostachys edulis* mixed forests, with decreasing relative abundance of Glomerales and increasing *Rhizophagus* with higher invasion degrees, though AMF diversity remains unaffected (Zou et al., 2023).

#### 4.6.5 Soil colony composition and structure

In invaded secondary forests by bamboo, the phospholipid fatty acid (PLFA, quantitatively reflecting the biomass and total biomass of different groups of living microorganisms in the sample) ratio of bacterial to fungal increases, while bacterial PLFA content in the organic layer decreases markedly (Wang et al., 2016). Comparing soil microbial communities in *Phyllostachys edulis* forests, nearby cedar plantations, and the transitional zone of bamboo invasion, *Phyllostachys edulis* invasion increases soil bacterial community diversity, with dominant bacteria remaining the same but differing in relative abundance (Lin et al., 2014).

The invasion also significantly alters bacterial and fungal community structures in *Cedrus deodara* plantation, reducing the proportion of Gram-positive and Gram-negative bacteria in total PLFA (Chang and Chiu, 2015). Post-invasion, the microbial community structure in subtropical broad-leaved forests shifts towards a lower fungi-to-bacteria ratio (F:B, the higher the ratio, the healthier the soil) (Zhao et al., 2021), increasing the diversity index (OTU abundance and Shannon index) of bacteria and fungi, and altering their community composition. The invasion of Actinobacteria and Basidiomycota decreases, while Ascomycota and Mortierellomycota increase (Liu C. et al., 2023). The abundance of *Acidobacteria* bacterium and *Acidobacteria* bacterium 13_2_20CM_58_27, and *Verrucomicrobia* bacterium decreases with lower *Phyllostachys edulis* invasion, while the abundance of *Alphaproteobacteria*, Actinobacteria bacterium, Trebonia kvetii, and *Bradyrhizobium erythrophlei* increase (Sun et al., 2023b).

#### 4.6.6 Intercropping effects

Due to the fast growth of bamboo, there is sufficient space under the forest for intercropping other plants. Intercropping other plants in bamboo undergrowth can alter the rhizosphere microbial composition and abundance. The rhizosphere soil of *Phyllostachys edulis* was studied after 2 years of intercropping with medicinal plants *Tetrastigma hemsleyanum*, *Paris polyphylla*, and *Bletilla striata* (Zhang et al., 2019b). The result showed that the Chao1, Ace, and Shannon indices of bamboo rhizosphere bacteria are significantly higher with *Paris polyphylla* intercropping compared to control *Phyllostachys edulis*. However, the α-diversity index of rhizosphere bacteria significantly decreases with *Bletilla striata* intercropping. In the fungal community, richness decreases when the medicinal plants are intercropped under *Phyllostachys edulis* forest. Intercropping with *Paris polyphylla* significantly reduces the relative abundance of Actinomycetes and Mucoromycota and increases the relative abundance of Gemmatimonadetes in *Phyllostachys edulis* rhizosphere soil. Compared to control, intercropping with *Tetrastigma hemsleyanum* significantly increases the relative abundance of Acidobacteria and Blastomonas in *Phyllostachys edulis* rhizosphere. Intercropping with *Bletilla striata* significantly increases the relative abundance of Acidobacteria and WPS-2 in *Phyllostachys edulis* rhizosphere. Intercropping with *Tetrastigma hemsleyanum* and *Bletilla striata* significantly reduces the abundance of Mortierellomycota in *Phyllostachys edulis* rhizosphere.

## 5 Perspective

The bamboo industry plays a crucial role in economic development, especially in subtropical regions like India, Japan, and south China. Compared to woody plants, bamboo grows rapidly and can supply raw materials to the industry at a higher frequency, thus accelerating product production. Rich bamboo germplasm resources and extensive cultivation areas provide a solid foundation for the development of the bamboo industry. However, promoting the productivity of bamboo forests and ensuring their healthy growth are inevitable challenges for the long-term sustainability of the bamboo industry.

Recent advances in bamboo genomics research have laid the groundwork for a deeper understanding of bamboo growth mechanisms and interactions with microorganisms. With new technologies, research on the relationships between bamboo plants and microorganisms has become more comprehensive. However, bamboo growth is somewhat limited by climate and other unfavorable factors. Therefore, exploring the interaction mechanisms between microbes and bamboo, and facilitating bamboo yield and growth, holds significant importance. Future research should delve into the following scientific and technical aspects of bamboo-microbe interactions:

Current research on bamboo-associated microorganisms is limited to a few bamboo species. It is essential to broaden the research scope through identifying bamboo plant-specific microbial strains and communities and developing their utilization value. For instance, the discovery of four new fungi genera in the *Phyllostachys edulis* seeds indicates the presence of bamboo-specific microorganisms. Exploring their functions, mechanisms, and application values is crucial.

The composition and structure of microbial communities can alter under certain treatments or environmental conditions. The functions of these communities and the regulatory mechanisms are not clearly and deeply analyzed beyond the observed changes. Additionally, current research methods restrict study of microbial composition and diversity. For instance, primer constraints during sequencing lead to limited microbial composition and fail to identify specific strains. Developing new methods to perform relative research according to the specific characteristics of genes from microorganisms is essential. Whole-genome sequencing of plants is a prerequisite for accurate metagenomic sequencing of endophytic bacteria. Currently, only a few bamboo species have completed whole-genome sequencing, limiting the widespread use of metagenomic sequencing to detect endophyte composition. Sequencing the whole genomes of various bamboo species will facilitate understanding of the gene constitution of bamboo plants and enhance the use of metagenomic sequencing to study microorganisms.

Further exploration is needed on how microbes regulate bamboo growth. Key questions include which secretions of bamboo plants recruit microbes to colonize the rhizosphere and surrounding soil, the gene expression patterns related to plant growth after microbial colonization, and whether there is a specific recruitment relationship between bamboo root exudates and microbes.

Growth-promoting bacteria play a positive role in crop (such as rice) growth and abiotic stress responses, have been widely used as bio-bacterial fertilizers in crops. However, similar studies on bamboo are scarce. Developing and studying growth-promoting bacteria isolated from bamboo, their colonization and proliferation characteristics, and their functions in alleviation stresses may provide new methods and insights for creating bio-bacterial fertilizers to enhance bamboo growth efficiency.

At last, these areas of research will not only contribute to the sustainable development of the bamboo industry but also offer new insights into plant-microbe interactions, potentially leading to innovative agricultural practices and products.
